# Ventricular Tachycardia or Not? Decoding Post-Cardioversion Wide Complex Tachycardia in a CRT-D Patient

**DOI:** 10.1016/j.jaccas.2026.108549

**Published:** 2026-07-29

**Authors:** Oroshay Kaiwan, Karthik Budharaju, Poornima Vinod, Bala Pushparaji, Maxine Nelson, Christopher Cunningham, Soufian AlMahameed

**Affiliations:** a Department of Internal Medicine, MetroHealth Medical Center, Cleveland, Ohio, USA; b Department of Cardiovascular Medicine, MetroHealth Medical Center, Cleveland, Ohio, USA

**Keywords:** AV nodal reentrant tachycardia, cardiac resynchronization therapy, electrocardiography, implantable cardioverter-defibrillator, supraventricular tachycardia, ventricular tachycardia, wide complex tachycardia

## Abstract

**Background:**

Wide complex tachycardia (WCT) in patients with structural heart disease is often presumed to be ventricular tachycardia (VT). However, supraventricular tachycardia with aberrant conduction accounts for a substantial proportion of WCT presentations and remains an important diagnostic consideration.

**Case Summary:**

A 74-year-old man with nonischemic cardiomyopathy and cardiac resynchronization therapy–defibrillator underwent cardioversion for persistent atrial fibrillation. Several hours later, he developed sustained WCT initiated by a premature ventricular complex. Although initially concerning for monomorphic VT, careful comparison with underlying baseline nonpaced electrocardiograms demonstrated nearly identical QRS morphology. Tachycardia terminated with 5 mg intravenous metoprolol.

**Discussion:**

This case highlights key electrocardiographic principles for differentiating VT from supraventricular tachycardia with aberrancy in patients with paced QRS, the diagnostic value of baseline QRS comparison, and important limitations of morphological Brugada criteria and device detection algorithms.

**Take-Home Message:**

Systematic underlying electrocardiogram analysis remains critical in the evaluation of WCT, to prevent inappropriate therapies.

## Visual Summary

**Figure:**
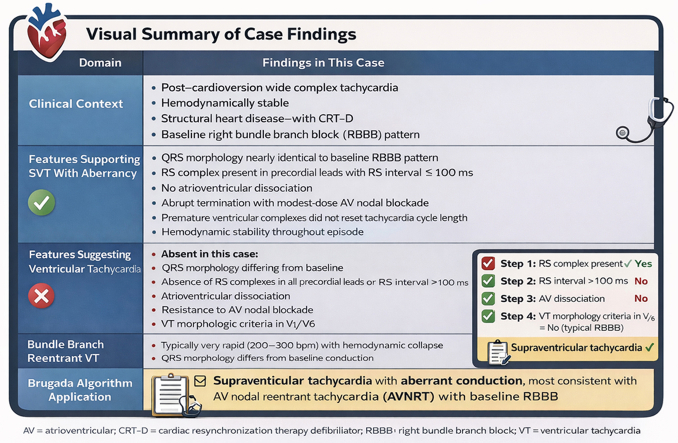

**Visual Summary:**
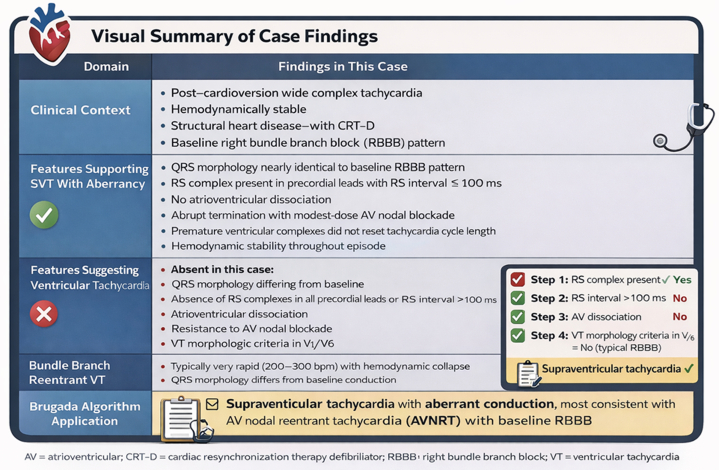
Application of Brugada Algorithm in Our Patient With WCT AV = atrioventricular; CRT-D = cardiac resynchronization therapy–defibrillator; VT = ventricular tachycardia.

## History of Presentation

A 74-year-old man presented with 1 week of progressive fatigue and lightheadedness. Physical examination revealed an irregular rate and rhythm of heart sounds, without any murmurs. Lungs were clear to auscultation, and there was no lower extremity edema.

**Take-Home Message**

• Systematic underlying electrocardiogram analysis remains critical in the evaluation of WCT, to prevent inappropriate therapies.

On presentation, the patient was hemodynamically stable, with unremarkable serum electrolytes and normal cardiac biomarkers. Device interrogation demonstrated persistent atrial fibrillation without recorded ventricular arrhythmias. Given his symptoms, elective electrical cardioversion was performed, successfully restoring normal sinus rhythm. A post-cardioversion electrocardiogram demonstrated an underlying intraventricular conduction delay (IVCD) with a right bundle branch block pattern (Figure 1).

**Figure 1:**
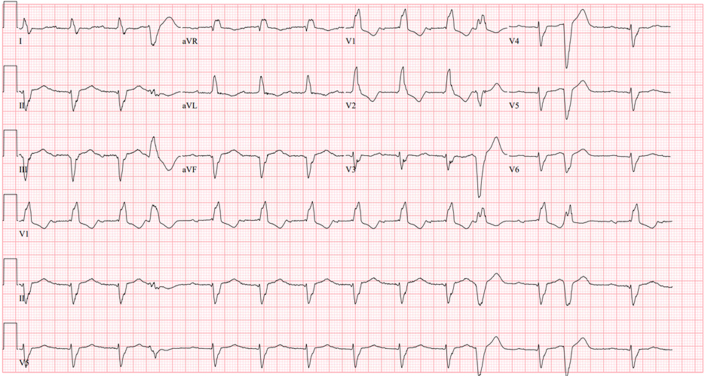
Electrocardiogram Showing Native Sinus Rhythm with Underlying Right Bundle Branch Morphology With Premature Ventricular Contractions

Several hours after cardioversion, the patient developed sustained wide complex tachycardia (WCT) lasting approximately 20 minutes, with a ventricular rate of approximately 140 beats/min (Figure 2). The arrhythmia was initiated by a premature ventricular complex (PVC). In addition, telemetry demonstrated an isolated PVC occurring during the tachycardia without resetting the cycle length (Figure 3).

**Figure 2:**
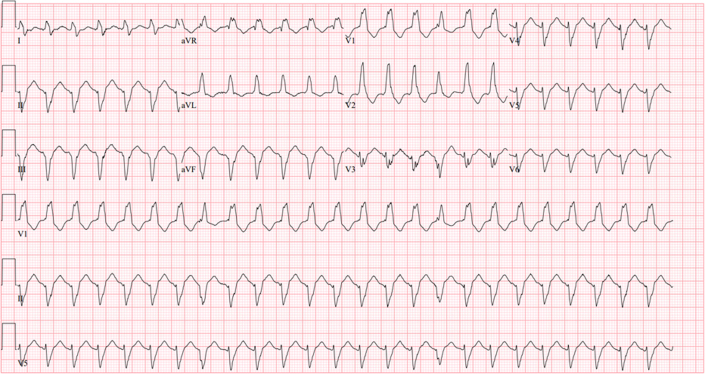
Electrocardiogram Showing Supraventricular Tachycardia With Aberrancy in the Setting of Underlying Right Bundle Branch Block

**Figure 3:**
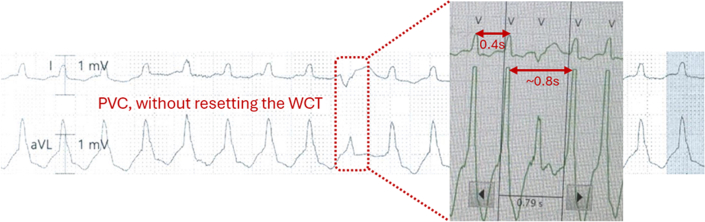
Telemetry Strip Showing a PVC Amidst the Tachycardia Premature ventricular complex (PVC) noted with a fixed tachycardia cycle length consistent with a reentrant mechanism. Frequent PVCs did not reset the tachycardia cycle length, which rules out ventricular tachycardia and orthodromic reciprocating supraventricular tachycardia, but can be seen in atrioventricular nodal reentrant tachycardia. WCT = wide complex tachycardia.

## Past Medical History

Past medical history included heart failure with reduced ejection fraction secondary to nonischemic cardiomyopathy (left ventricular ejection fraction 35%), status-post cardiac resynchronization therapy–defibrillator (CRT-D) implantation, persistent atrial fibrillation treated with metoprolol and amiodarone, hypertension, congenital bicuspid aortic valve status-post surgical aortic valve replacement, repaired aortic coarctation, nonobstructive coronary artery disease, prior ischemic stroke with residual right-sided deficits, and Crohn’s disease with colostomy.

## Differential Diagnosis

In the setting of structural heart disease and an implantable cardioverter-defibrillator (ICD), the sustained WCT was initially presumed to be ventricular tachycardia (monomorphic VT). Other diagnoses considered include supraventricular tachycardia (SVT) with aberrancy, antidromic atrioventricular (AV) reentrant tachycardia or SVT with QRS widening due to electrolyte abnormality or antiarrhythmic drugs.

## Investigations

A detailed evaluation was performed. Serum electrolytes and cardiac biomarkers earlier in the day were within normal limits. Prior electrocardiograms were reviewed, and careful inspection of the nonpaced surface electrocardiogram demonstrated that the QRS morphology during tachycardia closely matched the patient’s baseline QRS morphology during sinus rhythm. This finding favored a supraventricular origin with aberrant conduction.^1^

## Management

The patient remained hemodynamically stable throughout the episode. A single dose of intravenous metoprolol (5 mg) was administered, resulting in abrupt termination of the tachycardia and restoration of sinus rhythm.

## Outcome and Follow-Up

Following the episode, the patient remained in sinus rhythm. Subsequent CRT-D interrogation revealed that the tachycardia rate remained below the programmed ventricular detection threshold; therefore, no intracardiac electrograms were stored, and no antitachycardia pacing or ICD therapies were delivered.

## Discussion

We present a case of WCT in a patient with CRT-D and history of nonischemic cardiomyopathy and IVCD. We showcase the presenting arrhythmia as SVT despite the presence of morphological criteria consistent with VT. This case shows the importance of obtaining and examining the underlying native QRS morphology to determine the accurate mechanism of WCT in patients with structural heart disease.

Most patients with CRT devices have underlying IVCD, often of typical left bundle pattern. However, some patients have nonspecific IVCD due to underlying myocardial fibrosis and scaring. WCT in patients with cardiomyopathy is often due to VT.^2^

The present case illustrates several features that increase the risk of misclassification, including the presence of structural heart disease with CRT-D therapy and initiation of tachycardia by a PVC. In such patients, WCT is often presumed to represent scar-mediated VT, making careful underlying electrocardiographic comparison particularly important. SVT with aberrancy accounts for up to one-third of WCT presentations, even in patients with underlying structural heart disease.^3^

Comparison of QRS morphology during tachycardia with baseline sinus rhythm electrocardiograms is among the most reliable methods for differentiating SVT with aberrancy from VT.^1^ Identical QRS morphology strongly favors supraventricular origin, particularly in patients with preexisting conduction abnormalities.^1^ Response to AV nodal blockade provides additional diagnostic value. Although rare VTs may demonstrate sensitivity to beta-blockers, scar-mediated monomorphic VT typically does not terminate abruptly with modest doses of AV nodal–blocking agents.^2^^,^^3^ Given the patient’s hemodynamic stability, clear surface electrocardiographic features favoring SVT with aberrancy, and absence of recurrent arrhythmia or ICD therapy, invasive electrophysiologic confirmation was not clinically indicated. However, an electrophysiological study along with ablation of supraventricular pathway was planned in case of recurrence.

This case also underscores important limitations of ICD detection algorithms. Arrhythmias occurring below programmed therapy zones may not be recorded or treated, emphasizing that device interrogation alone may be insufficient for accurate rhythm diagnosis.^4^

Bundle branch reentrant ventricular tachycardia (BBRVT) also was considered in the differential diagnosis.^5^ BBRVT typically occurs in patients with cardiomyopathy with significant His-Purkinje conduction disease and is frequently associated with prolonged baseline His-ventricular intervals and ventriculoarterial dissociation. In this case, the QRS morphology during tachycardia was nearly identical to the baseline QRS morphology, there was no evidence of AV dissociation, no resetting of tachycardia despite many single PVCs at different coupling intervals during tachycardia, and the arrhythmia terminated with modest AV nodal blockade. These findings make BBRVT less likely and favor a supraventricular mechanism.

The Brugada algorithm provides a stepwise electrocardiographic approach to differentiate VT from SVT with aberrant conduction in patients presenting with regular WCT.^3^ Absence of an RS complex in all precordial leads (V_1_-V_6_) supports a diagnosis of VT, whereas the presence of an RS complex in any precordial lead suggests SVT with aberrancy and warrants further analysis.^3^ An RS interval >100 ms measured from the onset of the R wave to the nadir of the S wave in any precordial lead favors VT, whereas an RS interval ≤100 ms supports SVT with aberrant conduction.^1^^,^^3^ Evidence of AV dissociation strongly favors VT, although its absence does not exclude ventricular origin.^2^^,^^3^ When RS criteria are inconclusive, morphologic features in leads V_1_ and V_6_ consistent with ventricular activation favor VT, whereas typical bundle branch block patterns support SVT with aberrancy.^1^^,^^3^ If any step of the algorithm supports VT, the rhythm should be classified as VT; only when all VT criteria are absent should SVT with aberrancy be diagnosed.^3^

## Conclusions

In patients with CRT devices and regular WCT, it is imperative to compare the underlying QRS morphology with the tachycardia QRS morphology, even in the presence of morphological criteria favoring VT. Even in the era of advanced device therapy, fundamental electrocardiogram principles are essential to avoid inappropriate ICD interventions.^4^^,^^6^

## Funding Support and Author Disclosures

The authors have reported that they have no relationships relevant to the contents of this paper to disclose.
